# Metabolic engineering using iterative self-cloning to improve lipid productivity in ***Coccomyxa***

**DOI:** 10.1038/s41598-018-30254-7

**Published:** 2018-08-06

**Authors:** Yuki Kasai, Takuya Tsukahara, Fukiko Ikeda, Yoko Ide, Shigeaki Harayama

**Affiliations:** 1 Department of Biological Sciences, Faculty of Science and Engineering, Chuo University, Bunkyo-ku, Tokyo, 112-8551 Japan; 2 Research and Development Initiative, Chuo University, Bunkyo-ku, Tokyo, 112-8551 Japan; 3 Present Address: Advanced Research and Innovation Center, DENSO CORPORATION, Nisshin, 470-0111 Japan

## Abstract

We previously developed a self-cloning system that introduces cDNA of the uridine monophosphate synthase gene (*cUMPS*) of *Coccomyxa* sp. strain Obi as a selectable marker into uracil-auxotrophic mutants (Ura^−^) of the same alga. Here, we developed a Cre/*loxP*-based system for the removal of *cUMPS* flanked by directly repeated *loxP* sites from the *Coccomyxa* genome using the intracellular delivery of purified Cre recombinase to generate an Ura^−^ strain that was used as a host for second-round transformation using *cUMPS* as the selection marker. Employing this marker–gene-recycling system, *Coccomyxa* strains devoid of foreign DNA except the 34-bp *loxP* sequence, which overexpressed an acyl-(acyl-carrier-protein) thioesterase gene, and a type-2 diacylglycerol acyltransferase gene, were constructed by the sequential introduction of two expression cassettes for the respective genes. One of the resulting strains showed 1.4-fold higher lipid productivity than the wild-type strain. This method will be applicable to other eukaryotic microalgae to create marker-free transgenic strains.

## Introduction

Increase in greenhouse gas emissions and associated global warming has led to growing interest in the development and production of biofuels. Microalgae have long been recognized as a potential source for biofuel production because of high oil content, high growth rate, and non-requirement of arable land for cultivation^1^. However, the costs of algae-based fuels are currently not competitive with either petroleum or biodiesel made from vegetable oils, cooking oil waste, and animal fat. Thus, strain improvement to increase microalgal lipid productivity would be required. However, studies on this subject are still at very preliminary stages^2^.

Crop plants have a long history of breeding and have been genetically modified over the centuries. To further accelerate the rate of crop improvement, genetic engineering techniques have been used to introduce beneficial traits, such as herbicide tolerance and insect resistance. In the second generation of the techniques, traits were combined into a single crop to produce stacked traits^3^. On the other hand, genetically modified crops have also generated considerable controversy about the risks of transgenic technologies to human health and the environment. Thus, efforts for crop improvement without introducing foreign DNA have been made to avoid public concern. These methods are referred to as “self-cloning” in microorganisms and “cisgenesis and intragenesis” in plants. Cisgenesis is a genetic engineering method introducing “self-genes” (genes derived from the species itself or from a cross-compatible species) without changing its synteny, while intragenesis allows the introduction of recombinant DNA made of “self-genes”^4^.

We have been interested in applying recombinant DNA technologies in the unicellular oleogenic green alga *Coccomyxa* sp. strain Obi (formerly called *Pseudochoricystis ellipsoidea* strain Obi^5^; hereafter referred to as strain Obi) to improve its lipid productivity.

We developed a self-cloning system using an Ura^−^ derivative of strain Obi as a host and cDNA of the uridine monophosphate synthase gene (*cUMPS*) of the same strain as a selectable marker^5^. Using the system, we had tried, without success, to simultaneously introduce and overexpress two endogenous genes encoding acyl-[acyl-carrier-protein (ACP)] thioesterases (*FAT1*) and diacylglycerol acyltransferase 2 (*DGAT*2*d*), in strain Obi. The failure was attributed, at least in part, to two reasons. First, the frequency of transformants containing the intact expression units of the two genes was very low (less than 10% of Ura^+^); and second, the transgene expression was not always high probably due to transgene silencing. This problem could be overcome if the two expression cassettes are introduced sequentially, each time selecting the best transformant(s). However, our current self-cloning system with only one selectable marker gene is not applicable to such sequential introduction of transgenes.

Several site-specific recombinase-based systems have been developed to remove selectable markers from transgenic plants^6^. The most extensively used is the Cre/*loxP*-mediated site-specific DNA excision system from the bacteriophage P1. In this system, Cre recombinase specifically recognizes the 34-bp *loxP* sequence and excises a DNA segment flanked by two direct repeats of *loxP*, leaving a single copy of *loxP* (Supplementary Fig. S1)^7–9^. This system has been proven to be a powerful tool for creating marker-free transgenic organisms^10–12^. Kasai and Harayama^13^ reported that the Cre/*loxP*-mediated site-specific recombination system was applicable in the green unicellular alga *Chlamydomonas reinhardtii*.

In this study, we initially developed a method for the excision of the *cUMPS* selectable marker by the Cre/*loxP*-mediated site-specific recombination system in stain Obi. Subsequently, we sequentially introduced two expression cassettes, one for *FAT1* cDNA and another for *DGAT2d* cDNA, to create self-cloning transformants with enhanced lipid productivity.

## Results

### Construction of strain M2 carrying a single chromosomal copy of the *loxP_cUMPS_loxP* sequence

Strain M2 is a derivative of strain Obi with a defect in the gene for uridine monophosphate synthase (*UMPS*). The strain is thus the uracil auxotroph (Ura^−^) and resistant to 5-fluoroorotic acid (5-FOA^r^). The ploxP_cUMPS plasmid contains the cDNA of *UMPS* (*cUMPS*) flanked by the promoter and terminator of the *RBCS* gene encoding the small subunit of ribulose-1,5-bisphosphate carboxylase/oxygenase (the *cUMPS* expression cassette^5^). The *cUMPS* expression cassette in the ploxP_cUMPS plasmid is sandwiched by a direct repeat of the *loxP* site, and this structure is hereinafter referred to as *loxP_cUMPS_loxP* (Supplementary Fig. S2a). The ploxP_cUMPS plasmid was introduced into strain M2 by particle bombardment, and Ura^+^ transformants were selected on MA5 plates. The presence or absence of the *loxP_cUMPS_loxP* sequence in the genomes of 436 transformants was examined by PCR with the primer set, loxPF and loxPR (all the primer sequences used in this study are shown in Supplementary Table S1), and forty transformants were shown to contain the intact *loxP_cUMPS_loxP* sequence (Supplementary Fig. S2b). Seven (TT2-1, TT2-28, TT2-34, TT3-5, TT4-46, TT5-24, TT6-29, and TT7-24) of them were randomly selected to perform Southern blot analysis for the detection of NcoI restriction fragments containing a partial *cUMPS* sequence. Three NcoI fragments of 6.8 kb, 0.8 kb, and 3.5 kb, which correspond to the NcoI fragments of the endogenous *UMPS* (Supplementary Fig. S3a), were detected in the genomic DNA of strain M2. These three bands were detected in all seven DNA samples (Supplementary Fig. S3b). The NcoI restriction endonuclease cuts the ploxP_cUMPS plasmid once at the 5′-end of *cUMPS* (Supplementary Fig. S2a). Therefore, the number of bands excluding the bands from endogenous *UMPS* corresponds to the copy number of transgenic *cUMPS* insertion. As seen in Supplementary Fig. S3b, four transformants (TT2-34, TT4-46, TT5-24, and TT6-29) contained a single copy of *cUMPS*, hence a single copy of the *loxP_cUMPS_loxP* sequence, whereas the remainder carried multiple *cUMPS* copies. The copy number of *cUMPS* was also determined by quantitative real-time PCR (qPCR). The estimation of the *cUMPS* copy number by qPCR was comparable to the results of the Southern blot analysis (Supplementary Table S2). Strain TT4-46 was selected for further studies to demonstrate the excision of the *loxP*_*cUMPS_loxP* sequence catalyzed by Cre recombinase.

To map the insertion site of the *loxP_cUMPS_loxP* sequence in the chromosome of strain TT4-46, the flanking sequence of the insertion was determined by genome walking and the sequence was aligned to the draft genome sequence of strain Obi (unpublished). A DNA fragment of the ploxP_cUMPS plasmid containing the intact *loxP_cUMPS_loxP* sequence was integrated in an intergenic region between the gene for predicted importin-11 and that for translation initiation factor 3 subunit I (Supplementary Fig. S4a).

### Excision of the *loxP_cUMPS_loxP* sequence by Cre recombinase delivered by electroporation

The *cUMPS* marker is the only available selection marker for self-cloning-based transformation of *Coccomyxa* strains, and the removal of the *cUMPS* marker from the genome of an Ura^+^ derivative of strain M2 is indispensable for second-round introduction of unselectable genes of interest using *cUMPS* as the selection marker. We thus attempted to remove the *cUMPS* marker flanked by directly repeated *loxP* sites from the genome of strain TT4-46 using the intracellular delivery of hexahistidine-tagged Cre recombinase (Cre_CH) purified by the method described in the Methods section.

*In vivo* excision of the *loxP_cUMPS_loxP* sequence from the genome of strain TT4-46 by purified Cre_CH recombinase was examined in two independent experiments in which varying amounts of Cre_CH recombinase between 0.5 and 25 µg were delivered into strain TT4-46 by electroporation, and *cUMPS*-free derivatives of strain TT4-46 were selected on MA5 plates containing uracil and 5-fluoroorotic acid (5-FOA). Since Cre_CH recombinase-mediated site-specific recombination may occur at a very low frequency^13,14^, 5-FOA was used to select against *cUMPS*-positive cells as the UMPS enzyme converts 5-FOA into toxic 5-fluorouridine monophosphate causing cell death.

The excision of the *loxP_cUMPS_loxP* sequence in 5-FOA^r^ colonies was subsequently examined by using the primer set, TT4-46F and TT4-46R5, which amplifies either a 3.6-kb fragment from genomic DNAs without the excision event or a 0.5-kb fragment from genomic DNAs which underwent Cre recombinase-mediated excision (Supplementary Fig. S4a,b,c,d). The precise excision of the *loxP_cUMPS_loxP* sequence was then confirmed by sequencing the 0.5-kb PCR fragments. It was shown that 10 µg of Cre_CH recombinase was necessary and sufficient for marker excision with an efficiency of 1.4 × 10^−7^ cell^−1^ (Table 1). Thus, we established a method for the iterative genetic transformation of strain Obi without using foreign DNA.

**Table 1:** Excision efficiency of the *loxP_cUMPS_loxP* sequence by electroporated Cre recombinase.

Amount of Cre protein per electroporation (µg)	No of 5-FOA^r^ clones	No of precisely excised clones	Excision efficiency (cell^−1^)^a^
0	13	0	0
0.5	40	0	0
2	26	0	0
4	12	0	0
10	60	29	1.5 × 10^−7^
25	43	30	1.5 × 10^−7^

^a^The efficiency was calculated by the number of precisely excised clones divided by the number of cells used for electroporation.

### Improvement of lipid productivity in strain Obi by metabolic engineering using iterative self-cloning

**Figure 1:**
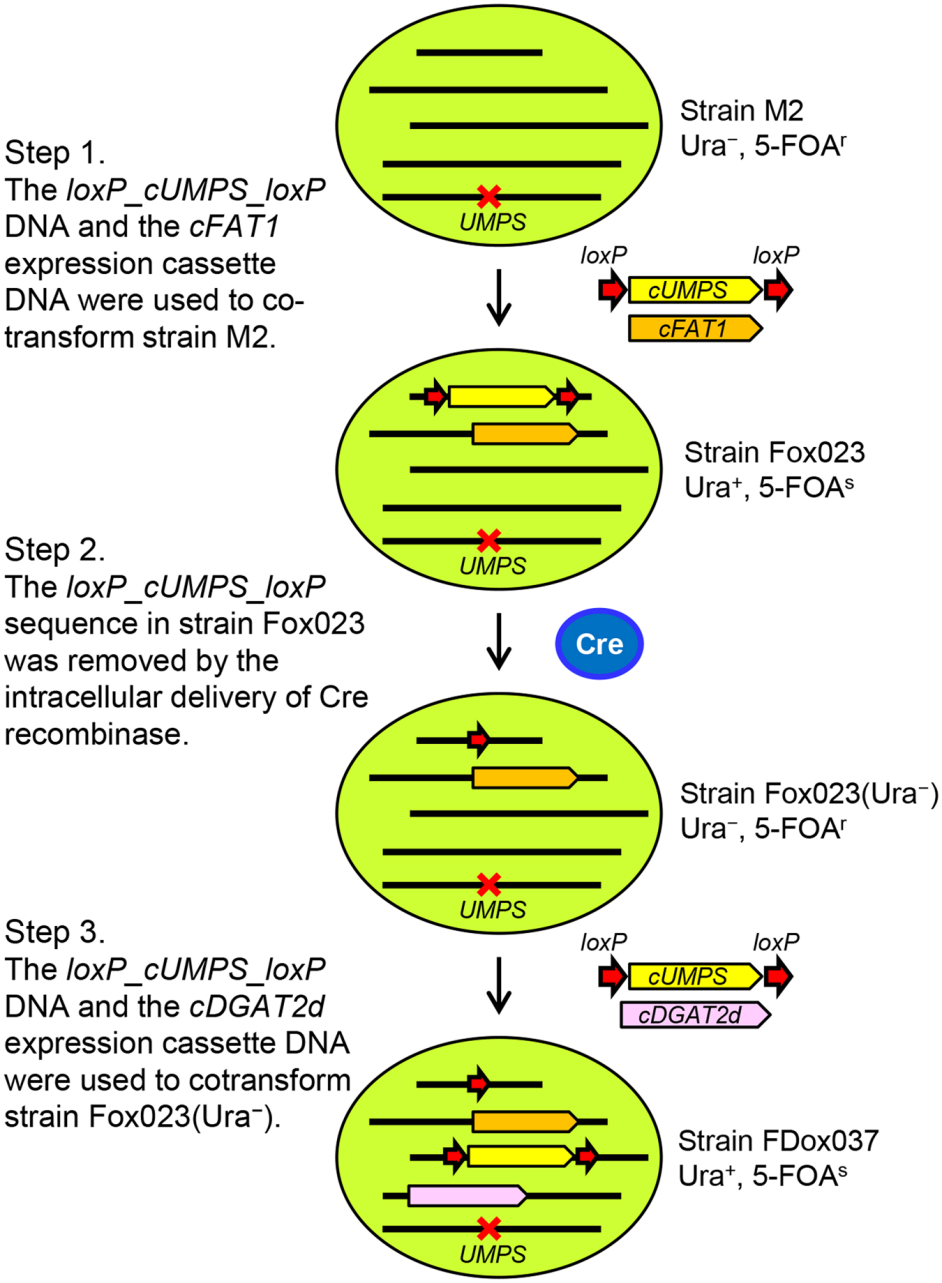
Three steps for the construction of self-cloning *Coccomyxa* overexpressing *cFAT1* and *cDGAT2d*. Step 1. The *loxP_cUMPS_loxP* DNA and the *cFAT1* expression cassette DNA were used to co-transform strain M2. Ura^+^ transformants, which carry a single copy of the *loxP_cUMPS_loxP* sequence and the intact *cFAT1* expression cassette sequence, were selected. Among the selected transformants, strain Fox023 exhibited the highest lipid productivity. Step 2. Purified Cre recombinase protein was introduced in cells of strain Fox023, and clones cured of the *loxP_cUMPS_loxP* sequence were identified among FOA^r^ derivatives. One of such clones named strain Fox023(Ura^−^) was used in step 3. Step 3. The *loxP_cUMPS_loxP* DNA and the *cDGAT2d* expression cassette DNA were introduced in strain Fox023(Ura^−^), and Ura^+^ transformants, which carry a single copy of the *loxP_cUMPS_loxP* sequence and the intact *cDGAT2d* expression cassette sequence, were selected. Among the selected transformants, strain FDox037 exhibited the highest lipid productivity.

We selected two endogenous genes, *FAT1* and *DGAT2d*, for overexpression in strain Obi because their expression is derepressed under nitrogen-depleted conditions or 150-mM NaCl administration; these two conditions enhance lipid biosynthesis (our unpublished results). We created self-cloning derivatives of strain Obi overexpressing the *FAT1* and *DGAT2d* cDNAs (c*FAT1* and c*DGAT2d*) in three steps (Fig. 1). First, the two DNA fragments, a 2.6-kb fragment containing the *cFAT1* expression cassette (Supplementary Fig. S5a) and a 3.3-kb fragment containing the *loxP_cUMPS_loxP* sequence flanked on both sides by partial sequences of the endogenous ubiquitin-like protein 5 gene (*UBL5*) (Supplementary Fig. S5c), were used to co-transform strain M2, and Ura^+^ transformants were screened on MA5 plates. The purpose to introduce the *loxP_cUMPS_loxP* sequence flanked by the partial *UBL5* sequences was twofold. In the *Coccomyxa* strains, the integration of introduced DNA fragment occurs, almost exclusively, via illegitimate recombination. If we introduce the *loxP_cUMPS_loxP* cassette without the flanking sequence, the majority of transformants would carry the cassette with a short deletion at both ends. Thus, the first purpose of introducing the flanking sequences was to increase the frequency of transformants harboring the intact *loxP_cUMPS_loxP cassette*. The second purpose was to facilitate the examination of precise excision of the *loxP-cUMPS-loxP* cassette in transformants: a PCR set to detect the precise excision was designed based on the flanking *UBL5* sequences.

A total of 312 Ura^+^ transformants were isolated, and the existence of the intact *cFAT1* expression cassette in the transformants was examined by PCR (Supplementary Fig. S5b). The intact *cFAT1* expression cassette sequence was detected in eight of the Ura^+^ transformants. The intact *loxP_cUMPS_loxP* sequence was also detected in the same eight Ura^+^ transformants (Supplementary Fig. S5d). qPCR analysis revealed that five out of the eight transformants (strains Fox023, Fox0232, Fox0929, Fox1115, and Fox1117) carried a single copy of the *loxP_cUMPS_loxP* sequence, whereas the remaining three strains carried multiple copies.

The expression of *cFAT1* in the five transgenic strains was analyzed using quantitative reverse transcription PCR (RT-qPCR) and normalized against 18S rRNA. The PCR primer set, FATqrtF and FATqrtR, detects cDNAs of both endogenous and transgenic transcripts of *FAT1*. Strains Obi and TT4-46 were used as *FAT1* expression controls. The *FAT1* expression was higher in strains Fox023 (10-fold), Fox0929 (4-fold), and Fox1115 (10-fold) than those in strains Obi and TT4-46 (Supplementary Fig. S6).

The effect of overexpression of c*FAT1* on the lipid productivity was examined in cells grown in 1/3 strength A7 medium for 14 days. The cellular lipid contents of strains Fox023 and Fox1115 were higher than those of strains Obi and TT4-46 (P < 0.01), whereas that of strain Fox0929 was lower than them. Although the lipid productivity of strain Fox023 was 1.1-fold higher than that of strain Obi, and 1.2-fold higher than that of strain TT4-46 (P < 0.01), that of strain Fox1115 was not higher than that of strain Obi or strain TT4-46 due to lower biomass yield of strain Fox1115 than any other strains (Table 2). Therefore, strain Fox023 was selected for further studies.

**Table 2:** Lipid productivity in *FAT1*-overexpressed strains.

Strain	Biomass concentration (g dry weight L^−1^)	Lipid content (%)	Lipid productivity (mg L^−1^ d^−1^)
Strain Obi	1.69 ± 0.06	38.8 ± 1.3	46.8 ± 1.4
TT4-46	1.62 ± 0.05	37.8 ± 0.7	43.8 ± 1.7
Fox023	1.71 ± 0.12	42.8 ± 1.5^**^	52.5 ± 4.8^**^
Fox0929	1.54 ± 0.04	36.4 ± 0.3	40.1 ± 1.2
Fox1115	1.42 ± 0.08	40.8 ± 0.7^**^	41.4 ± 2.8

The data are shown as mean ± standard deviation of triplicates.

The biomass dry weights and lipid contents were determined 14 days after the start of the cultivation. Statistical significance of differences between strain TT4-46 and the *FAT1*-overexpressed strains was tested by Student’s t-test (two tailed), and the results are shown as asterisks. Two asterisks indicate P < 0.01.

Before the introduction of the second transgene (*cDGAT2d*), the host strain Fox023 needed to be reverted to Ura^−^ by removing the *loxP_cUMPS_loxP* sequence. Thus, Cre_CH recombinase was electroporated into Fox023 as described in the previous subsection, and Fox023 derivatives resistant to 5-FOA were isolated. We found that strain Fox023 was more resistant to 5-FOA than its parental strain, and grew on MA5 plates containing uracil and 5.7-mM 5-FOA. Thus, we determined the minimal inhibitory concentrations against strain Fox023 by growing this strain on MA5 plates containing between 5.7-mM and 14.4-mM 5-FOA. Strain Fox023 did not grow significantly on MA5 plates containing either 11.5-mM or 14.4-mM 5-FOA. Thus, strain Fox023 derivatives no longer harboring *cUMPS* were selected on MA5 plates containing uracil and 11.5-mM 5-FOA.

**Figure 2:**
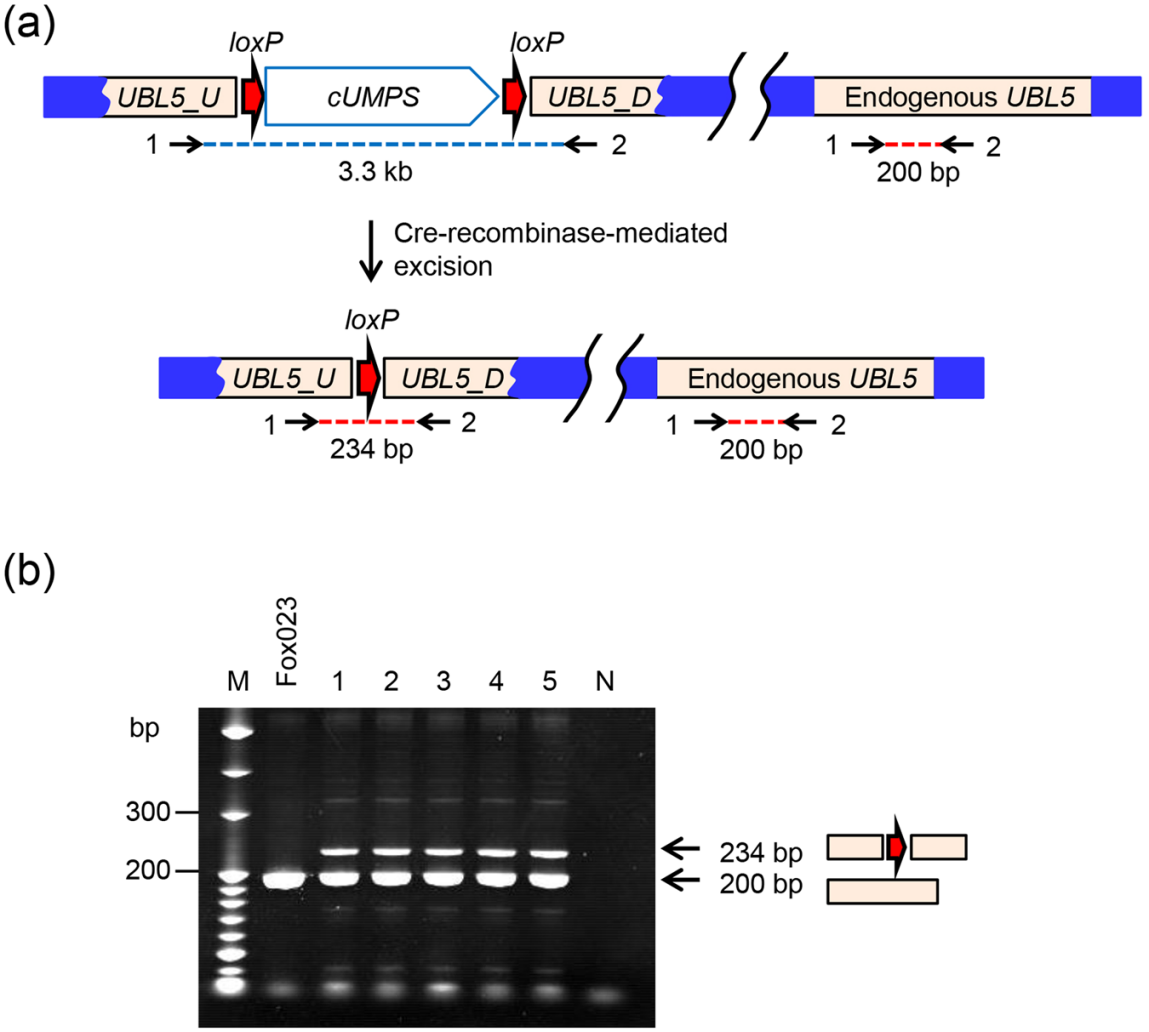
PCR analysis to detect the Cre-recombinase-mediated excision of the *loxP_cUMPS_loxP* sequence from strain Fox023. (**a**) The structure of the *loxP_cUMPS_loxP* sequence integrated in the chromosome of strain Fox023. The *loxP* sites are indicated as red arrows, the *UBL5* sequence as cream boxes, and irrelevant chromosome regions as blue boxes. PCR primers UBLF2 (**1**) and UBLR2 (**2**) were used for amplification of either a 3.3-kb fragment from genomic DNAs without the excision event or a 234-bp fragment from genomic DNAs which underwent Cre-recombinase mediated excision. A 200-bp fragment was also amplified from endogenous *UBL5*. (**b**) Agarose gel electrophoresis analyses of the 234-bp and 200-bp fragments amplified from genomic DNAs of indicated strains. Lane M: DNA size marker (20-bp DNA Ladder, TaKaRa) with molecular sizes in bp; Lane Fox023: strain Fox023; Lanes 1–5: five FOA^r^ and Ura^−^ derivatives which underwent Cre-recombinase mediated excision; Lane N: no template.

A total of 62 5-FOA^r^ colonies were obtained from three independent electroporation experiments with Cre_CH recombinase. The presence or absence of the *loxP_cUMPS_loxP* sequence was determined by PCR using primers UBLF2 and UBLR2. This primer set amplifies a 200-bp fragment from the endogenous *UBL5* gene, which was detected in all strains. A 234-bp band corresponding to the structure produced by Cre_CH recombinase-mediated excision of the *loxP_cUMPS_loxP* sequence was also amplified from five out of 62 5-FOA^r^ colonies (Fig. 2). The sequences of the 234-bp fragments were determined, and the results showed that the *loxP_cUMPS_loxP* sequence was precisely excised in the five derivatives, which were also Ura^−^, as expected. One of the 5-FOA^r^ and Ura^−^ derivatives was named Fox023(Ura^−^). A 3.3-kb fragment was amplified from the remaining 57 5-FOA^r^ colonies, which were Ura^+^, suggesting that they may be mutants showing low permeability to 5-FOA or high efflux pump activity. Thus, a 5-FOA concentration >11.5 mM should be used for clean selection of Ura^−^ derivatives from strain Fox023.

In the third step, a 2.6-kb fragment containing the *cDGAT2d* expression cassette (Supplementary Fig. S7a) and the 3.3-kb fragment containing the *loxP_cUMPS_loxP* sequence (Supplementary Fig. S7c) were introduced into strain Fox023(Ura^−^), and Ura^+^ transformants were screened on MA5 plates. The existence of the *cDGAT2d* expression cassette in 263 Ura^+^ transformants was then examined by PCR (Supplementary Fig. S7b). The intact *cDGAT2d* expression cassette sequence was detected in 19 Ura^+^ transformants. The intact *loxP_cUMPS_loxP* sequence was also detected in 14 among the 19 Ura^+^ transformants (Supplementary Fig. S7d). qPCR analyses revealed that five transformants (FDox013, FDox037, FDox129, FDox192, and FDox253) carried a single copy and nine carried multiple copies of the *loxP_cUMPS_loxP* sequence.

The expression of *cDGAT2d* in the five transgenic strains was analyzed using RT-qPCR and normalized against 18S rRNA (Supplementary Fig. S8a). The PCR primers DGATqrtF and DGATqrtR detect both the endogenous and transgenic *DGAT2d* transcripts. All strains showed >4.7-fold higher expression of *DGAT2d* transcripts than strain Fox023.

The five strains overexpressing both *cFAT1* and *cDGAT2d* were grown in 1/3 strength A7 medium for 14 days, and their lipid productivity was determined. The growth yield and cellular lipid content of strain FDox253 were significantly lower than those of the control strain (Fox023). Strain FDox253 thus has a slight metabolic disorder, which may have been caused by a transgene insertional mutation. In the remaining four transgenic strains, the levels of lipid content and growth yield were significantly higher in strains FDox037 and FDox129 than those of the control strains (Table 3, P < 0.01) whereas those of strains FDox013 and FDox192 were almost the same as those of strain Fox023. The increased TAG synthesis in strains FDox037 and FDox129 could be explained by the fact that the overexpression of *cFAT1* would provide more cytosolic acyl-CoA, and the overexpression of rate-limiting *cDGAT2d* would direct the metabolism of the cytosolic acyl-CoA to TAG formation. However, it is not clear how the simultaneous overexpression of *cFAT1* and *cDGAT2d* increased growth yield (Table 3).

**Table 3:** Lipid Productivity in *FAT1*- and *DGAT2d*-overexpressed strains.

Strain	Biomass concentration (g dry weight L^−1^)	Lipid content (%)	Lipid productivity (mg L^−1^ d^−1^)
Strain Obi	1.62 ± 0.04	38.8 ± 1.0	44.8 ± 1.2
TT4-46	1.65 ± 0.10	37.2 ± 0.3	43.7 ± 2.7
Fox023	1.65 ± 0.14	41.1 ± 1.2	49.6 ± 5.5
FDox013	1.64 ± 0.16	40.4 ± 0.9	47.5 ± 5.0
FDox037	2.07 ± 0.06	43.5 ± 1.0^**^	64.5 ± 3.1^**^
FDox129	1.75 ± 0.07	43.9 ± 1.1^**^	54.9 ± 3.1^*^
FDox192	1.70 ± 0.04	41.0 ± 0.5	49.7 ± 1.3
FDox253	1.14 ± 0.11	31.9 ±± 2.1	26.0 ± 4.3

The data are shown as mean ± standard deviation of more than triplicates.

The biomass dry weights and lipid contents were determined 14 days after the start of the cultivation. Statistical significance of differences between strain Fox023 and the *FAT1*- and *DGAT2d*-overexpressed strains was tested by Student’s t-test (two tailed), and the results are shown as asterisks. A single asterisk indicates a P-value between 0.01 and 0.05, and two asterisks indicate P < 0.01.

**Figure 3:**
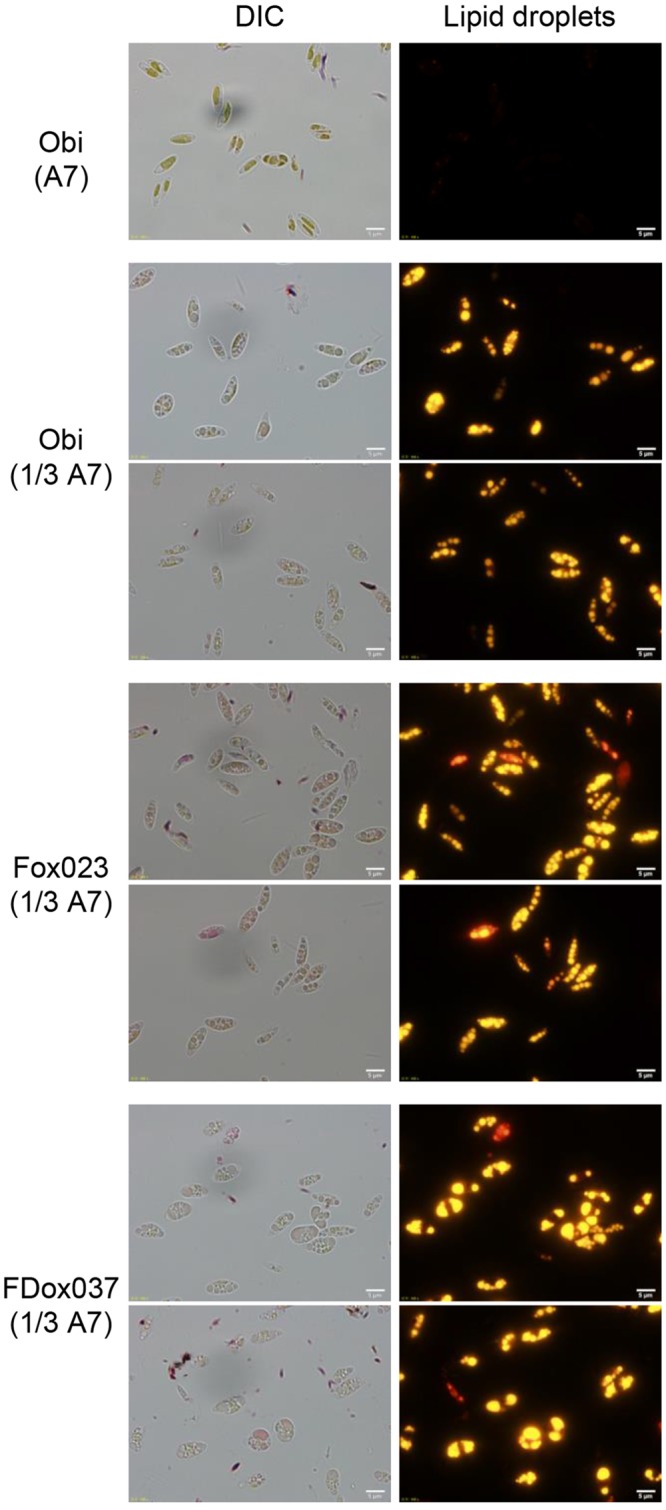
Accumulation of intracellular lipid droplets in transgenic *Coccomyxa* strains. Left panel, differential interference contrast (DIC) microscopy of cells; right panel, fluorescence microscopy of Nile Red-stained lipid droplets. All images were acquired with a fluorescent microscope (BX51, Olympus). The scale bar represents 5 μm. Cells were grown for 14 days in either A7 or 1/3 A7 medium.

Cells of strains Obi, Fox023 and FDox037 were grown in either 1/3 strength A7 medium or A7 medium, and they were stained with Nile Red, which is strongly fluorescent in neutral lipids. The micrographs in Fig. 3 showed that intracellular lipid droplets were detected as yellow fluorescent structures in cells grown in 1/3 A7 medium, but not in those grown in nitrogen replete A7 medium. The sizes of the lipid droplets examined with the ImageJ software were the largest in strain FDox037, followed by those in strain Fox023 and strain Obi (Supplementary Table S3). Size and dry weight of cell also varied among the three strains, those of strain FDox037 being the largest followed by those of strains Fox023 and Obi. The cell size and dry weight of strain Obi grown in A7 medium was smaller than those of strain Obi grown in 1/3 A7 medium.

Transgenic strains containing the *cDGAT2d* expression cassette but not the *cFAT1* expression cassette were constructed to compare lipid productivity levels with strain FDox037. The 2.6-kb fragment containing the *cDGAT2d* expression cassette and the 3.3-kb fragment containing the *loxP_cUMPS_loxP* sequence (Supplementary Fig. S7) were introduced into strain M2 to screen Ura^+^ transformants on MA5 plates. The existence of the *cDGAT2d* expression cassette in 383 Ura^+^ transformants was then examined in the same way as described before. The intact *cDGAT2d* expression cassette sequence was detected in 26 Ura^+^ transformants. The intact *loxP_cUMPS_loxP* sequence was also detected in eight of the 26 Ura^+^ transformants. qPCR analyses revealed that four transformants (strains Dox031, Dox262, Dox292, and Dox307) carried a single copy and four strains carried multiple copies of the *loxP_cUMPS_loxP* sequence.

The expression of *cDGAT2d* in strains Dox031, Dox262, Dox292, and Dox307 was analyzed using RT-qPCR and normalized against 18S rRNA. Three out of four strains (Dox262, Dox292, and Dox307) showed >12-fold higher expression of *cDGAT2d* transcripts than strain TT4-46 (Supplementary Fig. S8b).

The three strains overexpressing *cDGAT2d* were grown in 1/3 strength A7 medium for 14 days, and then lipid productivity was determined. The cellular lipid content of all strains was higher than those of strain Obi and strain TT4-46 (P < 0.05). The lipid productivity of strain Dox262 and strain Dox307 was 1.2- and 1.1-fold higher than strain TT4-46 (Supplementary Table S4, P < 0.05). These values were, however, lower than strains FDox037 and FDox129 overexpressing both *cFAT1* and *cDGAT2d* (Table 3). We thus inferred that the simultaneous overexpression of *cFAT1* and *cDGAT2d* synergistically enhanced the lipid productivity.

## Discussion

In this study, we demonstrated that some but not all transgenic *Coccomyxa* strains overexpressing FAT1 and/or DGAT2d showed improved lipid productivity. In fact, the correlation between the transgene expression and the lipid productivity in the transgenic strains was weak (Supplementary Fig. S6 vs Table 2; Supplementary Fig. S8a vs Table 3, and Supplementary Fig. S8b vs Supplementary Table S4). We infer that the weak correlation stemmed from two factors: (i) growth-phase-responsive expression of the transgenes, and (ii) variability in growth rates among independent Ura^+^ transformants. The transgenes used in this study were under the control of the *RBCS* promoter, whose expression decreased under nitrogen-deficient conditions (unpublished). Despite this, we determined the mRNA levels of the transgenes in cells from 7-day-old cultures because the quality of RNA extracted from nitrogen-starved cells in 14-day-old cultures was poor for qPCR analyses. Thus, the expression levels of the transgenes during the lipid-accumulating stage would certainly be much lower than those shown in Supplementary Figs S6 and S8. The primary purpose of the transgene expression analyses in this study was to exclude undesired transformants displaying transgene silencing from further analyses. Nevertheless, the lipid contents in the transgenic strains highly expressing *cFAT1* (strains Fox023 and Fox1115) were significantly higher than those of the control strains (Table 2 and Supplementary Fig. S6). Similarly, a good correlation (*R*^*2*^ = 0.95) was observed between the *cDGAT2d* expression levels and the lipid contents in *cDGAT2d* transformants (Supplementary Table S4 and Supplementary Fig. S8). These results corroborate our previous studies showing that the overexpression of *cFAT1* and *cDGAT2d* in *Coccomyxa* sp. strain KJ (a close relative of strain Obi) resulted in increased lipid accumulation (patent WO2017038993). Thus, we concluded that the overexpression of *cFAT1* and *cDGAT2d* in *Coccomyxa* cells was effective in increasing cellular lipid content.

On the other hand, the growth was slightly slower than for the parental strains in some Ura^+^ transformants (Table 2 and S4). Such phenomena were also observed in our previous study^5^. The reason for the slower growth of some Ura^+^ transformants could be either that the expression levels of *cUMPS* in the transformants were not optimum for growth, or that the insertion of exogenous DNA into their genomes disrupted gene(s) important for the rapid growth of cells.

Acyl-ACP thioesterase, the *FAT1* product, hydrolyzes acyl-ACP to release free fatty acids, which are subsequently activated by long-chain acyl-CoA synthases during export to the endoplasmic reticulum^14^. An increase in acyl-ACP thioesterase activity has been suggested to stimulate the export of free fatty acids from the chloroplast to the endoplasmic reticulum, where triacylglycerols (TAGs) are synthesized^15,16^. Recent reports have indicated that the overexpression of fatty acyl-ACP thioesterase from *Dunaliella tertiolecta* in *C. reinhardtii* increased TAG content^17^. Diacylglycerol acyltransferase 2, the *DGAT2d* product, catalyzes the final step of the canonical pathway for TAG biosynthesis (Kennedy pathway). The overexpression of endogenous *DGAT2* has been reported to enhance TAG accumulation in several microalgal species^18–21^. In this study, we demonstrated that the simultaneous overexpression of the two genes synergistically enhanced the lipid productivity. The increased lipid productivity in these strains could be explained by the fact that the overexpression of FAT1 would provide more cytosolic acyl-CoA, and the overexpression of rate-limiting DGAT2d would direct the metabolism of the cytosolic acyl-CoA to TAG formation. However, it is not clear how the simultaneous overexpression of FAT1 and DGAT2d increased growth yield (Supplementary Table S3), and more studies are required to clarify metabolic changes in these transgenic strains.

To the best of our knowledge, this is the first report on the molecular breeding of green algae using a self-cloning approach, and the first report of transgenic eukaryotic photosynthetic organism, which was constructed by an iterative introduction of two transgenes without introducing any foreign genes. This method will be applicable to produce marker-free transgenic strains of many other eukaryotic microalgae.

## Methods

### Algal strains and culture conditions

Strain M2, which is defective in *UMPS*, is a uracil-auxotrophic derivative of strain Obi^5^. Strain TT4-46, which carries a single copy of the *loxP_cUMPS_loxP* sequence, is an Ura^+^ derivative of strain M2. Strain Fox023, which carries a single copy of the *loxP_cUMPS_loxP* sequence and an intact *cFAT1* expression cassette, is a derivative of strain M2. Strain Fox023(Ura^−^), which is cured of the *loxP_cUMPS_loxP* sequence, is a derivative of strain Fox023. Strain FDox037, which carries a single copy of the *loxP_cUMPS_loxP* sequence and intact *cFAT1* and *cDGAT2d* expression cassettes, is a Ura^+^ derivative of strain Fox023(Ura^−^).

Strain Obi and its derivatives were cultured in 120-mL test tubes containing 50 mL of MA5 medium^22^ or MA5 medium supplemented with 1-mM uracil, in a plant-growth chamber (type #CLE-303, Tomy) under continuous illumination with daylight fluorescent tubes (40 W FL40S·FR·P, Panasonic) at 100 μmol photons m^−2^ s^−1^. To induce lipid biosynthesis under nitrogen-depleted conditions, cells were grown in 1/3 strength A7 medium containing 0.863-mM (NH_4_)_2_SO_4_, 2.38-mM urea, 0.26-mM KH_2_PO_4_, 0.26-mM K_2_HPO_4_, 0.4-mM MgSO_4_, 0.06-mM CaCl_2_, 0.4% (v/v) Fe solution (3 g L^−1^ citric acid, 4.9 g L^−1^ ammonium iron citrate, 0.5 g L^−1^ EDTA), 0.1% (v/v) trace element solution (70 mg L^−1^ H_3_BO_3_, 150 mg L^−1^ MnSO_4_·5H_2_O, 300 mg L^−1^ ZnSO_4_·7H_2_O, 300 mg L^−1^ CuSO_4_·5H_2_O, 70 mg L^−1^ CoCl_2_·6H_2_O, 3-mg L^−1^ Na_2_MoO_4_). Media was bubbled with 1% (v/v) CO_2_ at 25 °C. For agar-plate cultivation, MA5 medium was solidified using 1.5% agar (Bacto Agar or Noble agar, BD Difco). Agar plates were incubated in the plant-growth chamber.

### Construction of plasmids

Standard molecular procedures were performed as described by Sambrook *et al*.^23^. Genomic DNA was prepared as described by Imamura *et al*.^23^. PCR reactions for plasmid construction used PrimeSTAR Max DNA polymerase (Takara). All PCR primer sequences are listed in Supplementary Table S1.

The pUT2 plasmid carries the *cUMPS* expression cassette consisting of *RBCS*_P, *cUMPS*, and *RBCS*_T^5^. For the construction of the 3.2-kb-long *cUMPS* expression cassette flanked by the *loxP* sites (the *loxP_cUMPS_loxP* sequence), the corresponding fragment was amplified by PCR using primers loxPF and loxPR and pUT2 DNA as a template. The amplified fragment was digested using EcoRI and BamHI and inserted between the EcoRI and BamHI sites of the pBluescript II SK (+) plasmid to construct the ploxP_cUMPS plasmid (Supplementary Fig. S2a).

The *CRE* gene is a synthetic codon-optimized gene encoding Cre recombinase. The *CRE* gene was cloned into the BamHI and HindIII site of the pUC57 vector to create the pUCre plasmid.

For the construction of pCreCH, a 1.0-kb *CRE* fragment was amplified using primers Cre_HTF and Cre_HTR with pUCre DNA as a template, and the resulting fragment was purified using a PCR purification kit (Qiagen). The fragment was digested using NcoI and XhoI and inserted between the NcoI and XhoI sites of pET28a (+) (Novagen) to create pCreCH.

The ploxP_cUMPS_2 plasmid which carries the *loxP_UMPS_loxP* cassette flanked by the 5′ and 3′ regions of the ubiquitin-like protein 5 gene (*UBL5*) (Supplementary Fig. S7c) was constructed as follows. First, a partial endogenous ubiquitin-like protein 5 gene (*UBL5*) sequence was PCR-amplified using primers UBLF and UBLR and genomic DNA of strain Obi as a template. The amplified fragment was cloned into the HincII site of the pBluescriptII SK (+) plasmid using the Mighty Cloning Reagent (Blunt End) (Takara) to construct the pUbl5 plasmid. The DNA sequence was verified using double-stranded sequencing via a commercial service provider (Macrogen Japan Corp.). The *loxP_cUMPS_loxP* sequence fragment was PCR-amplified using primers loxPF2 and loxPR2 and ploxP_cUMPS plasmid DNA as a template. The amplified fragment was inserted into the DraI site in the first intron of *UBL5* in the pUbl5 plasmid to create ploxP_cUMPS _2.

To construct the pFAT1 carrying the *cFAT1* expression cassette (Supplementary Fig. S5a) and pDGAT2d plasmids carrying the *cDGAT2d* expression cassette (Supplementary Fig. S7a), total RNA was extracted from cells of strain Obi grown in 1/3 strength A7 medium to a density of OD_750_ = 4.0 using a TRIzol® plus RNA purification kit (Ambion). Residual DNA was digested with PureLink DNase (Ambion) according to the manufacturer’s instructions. First strand cDNA was synthesized using a Transcriptor High Fidelity cDNA Synthesis kit (Roche) with an anchored-oligo (dT)_18_ primer according to the manufacturer’s instructions. The cDNAs of *FAT1* and *DGAT2d* were amplified from a cDNA library using PCR with the primer sets FAT1F plus FAT1R and DGAT2dF plus DGAT2dR, respectively. The resulting amplified cDNAs were inserted into a pHA1101 plasmid digested with NcoI and ClaI with the In-Fusion HD EcoDry Cloning kit (Clontech). The sequences of the resulting *cFAT1* and *cDGAT2d* were verified using double-stranded sequencing.

### Transformation of strain Obi and its derivatives

Two methods were used for transformation: particle bombardment and electroporation. Particle bombardment was performed as described previously^5^. Following bombardment, cells were incubated at 25 °C in the dark for 24 h, and then transferred onto appropriate selection plates. Electroporation was performed as follows. Cells were grown under 12-h light/12-h dark cycles at a light intensity of 100 µmol m^−2^ s^−1^ in MA5 medium supplemented with 0.6-M sorbitol for three days until the density of OD_750_ reached 1.0. Cells were harvested by centrifugation at 2,900×*g* for 5 min, washed twice with 5-mM 2-morpholinoethanesulfonic acid (MES) buffer (pH 5.5) twice, and resuspended in Max Efficiency® Transformation Reagent for Alga (Thermo Fisher) to a final density of 1–2 × 10^9^ cells mL^−1^. DNA or protein was added to an aliquot of cell suspension and transferred to a pre-chilled 2-mm gap electroporation cuvette (Bio-Rad) and incubated at 16 °C for 2 min. An electric pulse of 10,000 V/cm with exponential decay was applied to the suspension with a GenePulser XCell^TM^ (Bio-Rad) with capacitance set at 25 µF and resistance set at 200 ohm. After electroporation, cells were incubated on ice for 10 min and transferred to 2 mL of MA5 medium containing 55-mM glucose. After incubation for 24 h at 25 °C under dim light, cells were transferred onto appropriate selection plates.

The ploxP_cUMPS plasmid containing the *loxP_cUMPS_loxP* sequence was introduced into strain M2 and Ura^+^ transformants were selected on MA5 plates prepared with Noble agar after incubation in a 5% (v/v) CO_2_ incubator at 25 °C under fluorescence tubes at 50 μmol photon m^−2^ s^−1^. The intact *loxP_cUMPS_loxP* sequence was then detected in lysates of the Ura^+^ transformants by PCR using primers loxPF3 and loxPR3.

A transgenic strain containing a single copy of the *loxP_cUMPS_loxP* sequence and the *cFAT1* expression cassette was constructed as follows. A 2.6-kb fragment containing the *cFAT1* expression cassette was amplified by PCR using primers RBCSPF2 and RBCSTR2 and pFAT1 DNA as a template. A 3.3-kb fragment containing the *loxP_cUMPS_loxP* sequence flanked on both sides by the endogenous *UBL5* sequence was amplified by PCR using primers UBLF2 and UBLR2 and ploxP_cUMPS _2 DNA as a template. These two DNA fragments consisting solely of endogenous DNA of strain Obi with the exception of the 34-bp *loxP* sites were introduced into strain M2, and Ura^+^ transformants were isolated on MA5 plates solidified with Noble agar as described above. The presence or absence of the *cFAT1* expression cassette in the Ura^+^ transformants was examined by PCR using RBCSPF or FAT1F as a forward primer and FAT1R or RBCTR as a reverse primer.

Transgenic strain containing a single copy of the *loxP_cUMPS_loxP* sequence and the *cDGAT2d* expression cassette was constructed as follows. Strain Fox023(Ura^−^) is an Ura^−^ strain harboring the intact *cFAT1* expression cassette. For the introduction of the *cDGAT2d* expression cassettes in either strain Fox023(Ura^−^) or strain M2, a 2.6-kb fragment containing the *cDGAT2d* expression cassette was PCR-amplified using primers RBCSPF2 and RBCSTR2 and the pDGAT2d plasmid DNA as a template. In addition, the 3.3-kb fragment containing the *loxP_cUMPS_loxP* sequence flanked on both sides by the *UBL5* sequence was amplified as described earlier. These two fragments were introduced into either strain Fox023(Ura^−^) or strain M2, and Ura^+^ transformants were isolated on MA5 plates solidified with Noble agar. The presence or absence of the *cDGAT2d* expression cassette in the Ura^+^ transformants was performed using RBCSPF or DGAT2dF as a forward primer and DGAT2dR or RBCTR as a reverse primer.

### Southern blot analysis

Five µg of genomic DNA from strain Obi or its derivatives, extracted as previously described^22^, was digested with NcoI, separated on a 0.7% (w/v) agarose gel, and blotted onto a Hybond-N+ membrane (GE Healthcare, UK) by standard capillary transfer in 20X SSC transfer buffer. The membrane was baked at 80 °C for 2 h. A *cUMPS* fragment was labeled using a DIG High Prime DNA labeling and detection kit (Roche). Hybridization and signal detection were performed according to the manufacturer’s instructions.

### Quantitative real-time PCR

qPCR using the comparative threshold cycle method (ΔΔCt)^24,25^ was performed on a Thermal Cycler Dice® real-time system II (TaKaRa) using the SYBR® Premix Ex Taq^TM^ II (Tli RNaseH Plus; TaKaRa) kit. For the determination of the *cUMPS* copy number, primers UqrtF and UqrtR were used. These primers detect both endogenous and transgenic *UMPS*. However, only a 150-bp PCR product from the transgene but not a 450-bp product from the endogenous gene was expected be amplified because of the very low amplification efficiency of the kit for fragments larger than 300 bp according to the instruction manual of the kit. Primers TqrtF and TqrtR were used for the amplication of *TUBA* encoding α-tubulin (*TUBA*). The amplification efficiencies of *cUMPS* and *TUBA* were calculated on the basis of the slope of the standard curves and were approximately 101% and 103%, respectively. Because the copy number of the *TUBA* gene per genome is one, a change in Ct value difference (ΔCt) corresponds to a change in copy number of *cUMPS*. By comparing the ΔCt value of the unknown samples to the ΔCt of the known control (strain TT4-46), ΔΔCt was obtained. Copy number can then be calculated using the equation: Copy number = 2^−ΔΔCt^. The melting curves for each PCR product were determined by measuring the decrease in fluorescence with increasing temperature from 60 °C to 95 °C. Each sample was analyzed in triplicate.

### Determination of the flanking DNA sequences of the *loxP_cUMPS_loxP* insertion in strain TT4-46

DNA sequences flanking the *loxP_cUMPS_loxP* insertion were determined using a Universal GenomeWalker 2.0 (Clontech) and Advantage 2 PCR kit (Clontech) by the step-down PCR protocol. The resulting amplified fragments were purified with the NucleoSpin Gel and PCR Clean-Up kit (Clontech) and cloned into the pGEMT-Easy plasmid (Promega). The nucleotide sequences were determined as described previously, and compared with the draft genome sequence of strain Obi.

### Expression in *Escherichia coli* and purification of Cre_CH recombinase

The plasmid pCreCH was transformed into BL21(DE3) (New England Biolaboratories) for protein expression. The bacterial culture was grown to an OD_600_ of 0.6, and the synthesis of Cre_CH recombinase was induced with 1-mM IPTG. The hexahistidine-tagged Cre_CH recombinase was purified with a Ni-NTA Fast Start kit (QIAGEN) under native conditions. The final eluent was buffer exchanged to phosphate-buffered saline containing 20% (v/v) glycerol with 10 K Amicon centrifugal filters (Millipore) and stored at −80 °C. Protein concentration was determined by Bradford Assay^26^, and SDS-PAGE was performed as previously described^27^. Purified Cre_CH recombinase was assayed *in vitro* as described by Lin *et al*.^28^, with the exception of using pLox2+^29^ as the substrate DNA. One unit of enzyme produces 10^4^ colonies (equivalent to 2 × 10^6^ circular molecules) in a 30-minute reaction with 200 ng DNA substrate suspended in 20 µL of 50-mM Tris-HCl buffer (pH 7.5) containing 33-mM NaCl and 10-mM MgCl_2_. The purified Cre_CH recombinase showed a specific activity of 1.7 × 10^4^ U (mg protein)^−1^, corresponding to 71% of the native Cre recombinase activity^30^.

### Quantitative reverse transcription PCR (RT-qPCR)

To estimate expression levels of transgenes, total RNA was extracted from cells grown in 1/3 strength A7 medium for 7 days to a density of OD_750_ = 2.0, as described previously. First strand cDNA was synthesized using a PrimeScript™ RT reagent kit with gDNA Eraser (Perfect Real Time, TaKaRa) and an RT primer mix containing oligo (dT)_18_ and random hexamers. To quantify *FAT1*and *DGAT2d* transcripts, RT-qPCR was performed using primers FATqrtF plus FATqrtR and DGATqrtF plus DGATqrtR, respectively. The levels of 18S rRNA were also determined as an internal control using primers 18SrRNAqrtF and 18SrRNAqrtR. The melting curves for each PCR product were determined as described above. RT-qPCR without a template was also performed in each experiment as a negative control.

### Determination of cellular lipid contents

To quantify lipid contents of strain Obi and its derivatives, cells grown under nitrogen-depleted conditions were collected by centrifugation and freeze-dried. Cellular neutral lipid content (mg lipid per mg dry weight of cells) in a sample of about 20 mg of freeze-dried cells was determined by time-domain Nuclear Magnetic Resonance (TD-NMR) using a benchtop low-resolution pulsed NMR instrument (model MQC; Oxford Instruments) with MultiQuant software (Oxford Instrument)^31,32^ in accordance with the ISO 10565 guideline^33^ using olive oil as a standard. Neutral lipid contents determined by the TD-NMR method in *Chlorella protothecoides*^34^ and *Coccomyxa* strains (our unpublished results) showed good agreement with those determined by the gravimetric method. Lipid content per liter of culture (mg lipid/L) was calculated by multiplying biomass concentration (mg dry weight of cells per liter of culture) by cellular lipid content. Because cellular lipid content at day 0 was almost zero, average daily lipid productivity at day X (mg lipid per liter of culture per day) was calculated by the equation: [(biomass concentration at day X–biomass concentration at day 0) × (cellular lipid content at day X)] ÷ X.

Intercellular lipid droplets were visualized by staining 80 µL of cell suspension with 20 µL of 500 μg mL^−1^ Nile Red fluorescent dye (Wako) dissolved in 100% (v/v) dimethyl sulfoxide. The stained cells were observed with a fluorescent microscope (BX51; Olympus) and image data were analyzed using the ImageJ 13.0 software (http://www.jmol.org/).

### Accession code

Sequence data from this study can be found in the DDBJ/NCBI data libraries under the accession numbers LC332837 (*DGAT2d* CDR), LC332838 (*FAT1* CDR), LC332839 (*UBL5* CDR), LC332840 (*TUBA* CDR), LC332841 (ploxP_cUMPS), LC332842 (ploxP_cUMPS_2), and LC332843 (pCre).

## Electronic supplementary material

Supplementary Information
