# Supplementary material for: Metabolic engineering using iterative self-cloning to improve lipid productivity in Coccomyxa
Source: Sci Rep. 2018 Aug 6;8:11742. doi: 10.1038/s41598-018-30254-7 (PMC6078956; doi:10.1038/s41598-018-30254-7)
Supplement: Supplementary file 1 — Supplementary Information [file 41598_2018_30254_MOESM1_ESM.pdf]

Supplementary Data

Metabolic engineering using iterative self-cloning to improve lipid productivity in *Coccomyxa*

Yuki Kasai[1,2*], Takuya Tsukahara[1], Fukiko Ikeda[1], Yoko Ide[1,2,3], and Shigeaki Harayama[1,2]

[1]Department of Biological Sciences, Faculty of Science and Engineering, Chuo University, Bunkyo-ku, Tokyo 112-8551, Japan

[2]Research and Development Initiative, Chuo University, Bunkyo-ku, Tokyo 112-8551, Japan

[3]Present address: Advanced Research and Innovation Center, DENSO CORPORATION, Nisshin, 470-0111, Japan

*Correspondence: ykasai@kc.chuo-u.ac.jp

Postal address: Department of Biological Science, Chuo University

Kasuga 1-13-27, Bunkyo-ku, Tokyo 112-8551

Supplementary Tables

Supplementary Table S1 — Primers used in this study

Primer	Sequence (5′ to 3′)
**For plasmid construction**	
**loxPF**	ACCGAATTCATAACTTCGTATAGCATACATTATACGAAGTTATATGCCCTCAATCGGCCGATT
**loxPR**	TTGGATCCATAACTTCGTATAATGTATGCTATACGAAGTTATTGTGCTGGTCTGTTTCCATG
**loxPF2**	ATAACTTCGTATAGCATACATTATACGAAGTTATATGCCCTCAATCGGCCGATT
**loxPR2**	ATAACTTCGTATAATGTATGCTATACGAAGTTATTGTGCTGGTCTGTTTCCATG
**UBLF**	ATGATTGAAGTGATTCTCAA
**UBLR**	CTAGTTGTAGTACAGCTCCA
**Cre_HTF**	GAAGGAGATATACATATGAGCAACCTGCTGACCGTGCAC
**Cre_HTR**	GTGATGGTGGTGATGATGGTCGCCGTCCTCCAGCAGGCG
**FAT1F**	TTGCTTTGTGCAGCCATGTCAATGTTCAGTACAAT
**FAT1R**	GTCGACGGTATCGATTCAGTGAAGCTGCAGTTTGC
**DGAT2dF**	TTGCTTTGTGCAGCCATGGCAGACTCATTCTGGAGT
**DGAT2dR**	GTCGACGGTATCGATTCACACTATCTGGAGCTCCT
**For the detection of transgenes and marker-excision event**	
**loxPF3**	GAATTCATAACTTCGTATAGCATAC
**loxPR3**	GGATCCATAACTTCGTATAATGTAT
**RBCSPF**	ACGGATCCGCGGCCGCTCTAGAACTAGTATGCCC
**RBCSTR**	ACAAGCTTTCGAGTGTGCTGGTCTGTTTCCATGCAG
**RBCSPF2**	CTCAATCGGCCGATTTCATGCATGA
**RBCSTR2**	GTGCTGGTCTGTTTCCATGCAGTCAT
**TT4-46F**	TAACGCTTACAATTTCCATTCGCCA
**TT4-46R5**	CGCAATTAATGTGAGTTAGCTCACT
**UBLF2**	CTTTGTACTTGTGCAACCGAGC
**UBLR2**	TCTGAATGCGAATCTTCTCTGGG
**For RT-qPCR**	
**UqrtF**	TTCATAGCCGCCAACCAGAC
**UqrtR**	CGCCATCAGCTCCAACAG
**TqrtF**	GACTCGCAAAACATCAGTGC
**TqrtR**	TGTTCAGCATACCCACACAC
**FATqrtF**	TCAGGTTTGTGCACATGCTG
**FATqrtR**	TCAGTGAAGCTGCAGTTTGC
**DGATqrtF**	TTGTTTGCAGCTGTCGTGTG
**DGATqrtR**	TGTATGCCCACCAGATATTGCC
**18SrRNAqrtF**	GGATCAATTGGAGGGCAAGT
**18SrRNAqrtR**	GCCCGAAATCCAACTACGAG

Supplementary Table S2 — *cUMPS* copy numbers estimated by Southern blot and RT-qPCR analyses

Strain name	Copy number estimated by Southern blot	Copy number estimated by qPCR
M2	0	0
TT2-1	2	2
TT2-28	3	3
TT2-34	1	1
TT3-5	2	2
TT4-46	1	1
TT5-24	1	1
TT6-29	1	1
TT7-24	9	8

Supplementary Table S3 — Sizes of cell and lipid body of strain Obi and its derivatives

Strain	Medium	Cell size (µm)[a]		Total lipid droplet area (μm^2/cell)[a]	Cell dry weight (pg/cell)[b]
		Length	Width		
Obi	A7	7.0 ± 0.7	2.0 ± 0.5	-	-
Obi	1/3 A7	7.8 ± 0.6	2.9 ± 0.7	9.8 ± 4.9	13.6 ± 1.8
Fox023	1/3 A7	8.4 ± 0.8	3.4 ± 0.3	10.6 ± 6.5	15.8 ± 0.6**
FDox037	1/3 A7	8.8 ± 1.3	4.1 ± 0.5**	15.7 ± 8.4**	18.4 ± 0.7**

Strains Obi, Fox023 and FDox037 were grown for 14 days in either A7 or 1/3 A7 medium. Statistical significance of differences between strain Obi (1/3 A7) and other strains was tested by Student's *t*-test (two tailed), and the results are shown as asterisks. A single asterisk indicates a P-value between 0.01 and 0.05, and two asterisks indicate $P < 0.01$.

[a]The data are shown as mean ± standard deviation of 40 cells.

[b]The data are shown as mean ± standard deviation of more than triplicates.

Supplementary Table S4 — Lipid productivity in *DGAT2d*-overexpressed strains

**Strain**	**Biomass concentration (g dry weight L^{-1})**	**Lipid content (%)**	**Lipid productivity (mg L^{-1} d^{-1})**
Strain Obi	1.76 ± 0.02	37.7 ± 0.2	47.4 ± 0.4
TT4-46	1.64 ± 0.08	37.6 ± 0.6	43.9 ± 2.3
Dox262	1.77 ± 0.06	41.3 ± 0.4**	52.1 ± 2.1**
Dox292	1.53 ± 0.05	38.6 ± 0.3*	42.1 ± 1.5
Dox307	1.58 ± 0.04	43.8 ± 0.3**	49.5 ± 1.5*

The data are shown as mean ± standard deviation of more than triplicates.
The biomass dry weights and lipid contents were determined 14 days after the start of the cultivation. Statistical significance of differences between strain TT4-46 and the *DGAT2d*-overexpressed strains was tested by Student's *t*-test (two tailed), and the results are shown as asterisks. A single asterisk indicates a P-value between 0.01 and 0.05, and two asterisks indicate $P < 0.01$.

Supplementary Figures

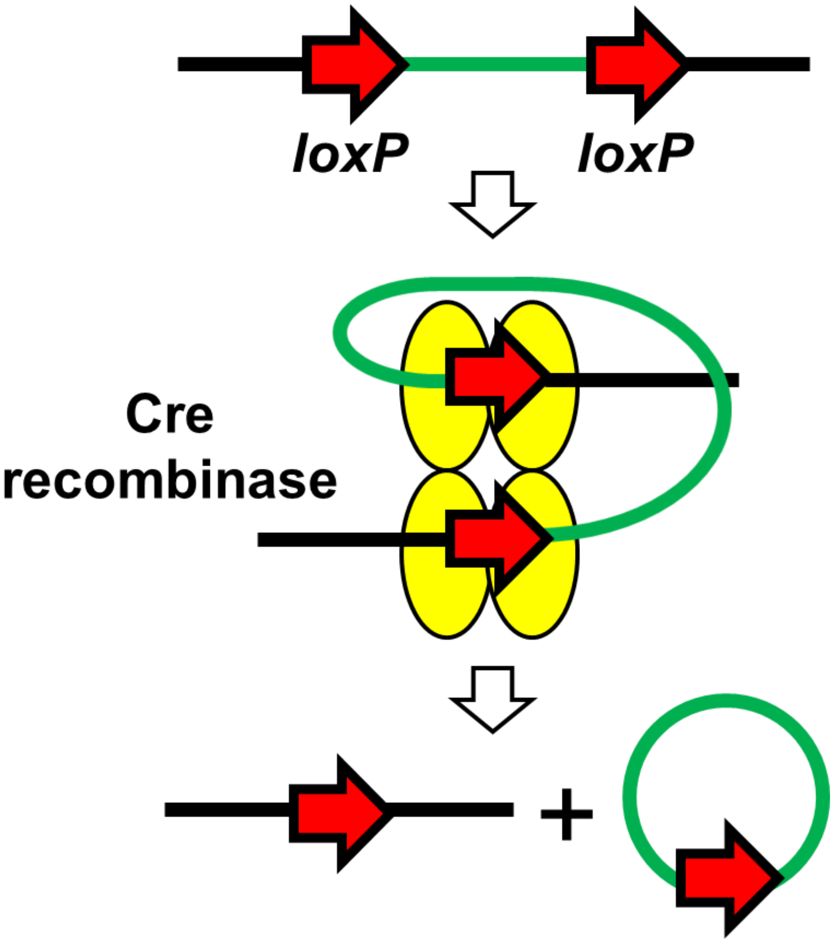

Supplementary Figure S1. The Cre/*loxP* system

The Cre/*loxP* system, which is originally found in bacteriophage P1, consists of the Cre recombinase (yellow ellipses) and a pair of the 34-bp-long *loxP* site (red arrows) flanking an intervening gene (green line) located on a chromosome (black line). The Cre recombinase recognizes the *loxP* sites, and catalyzes site-specific recombination between the *loxP* pair. As a consequence of the recombination, the intervening gene is excised to form a circular DNA carrying a single copy of *loxP*, leaving a single copy of *loxP* on the chromosome.

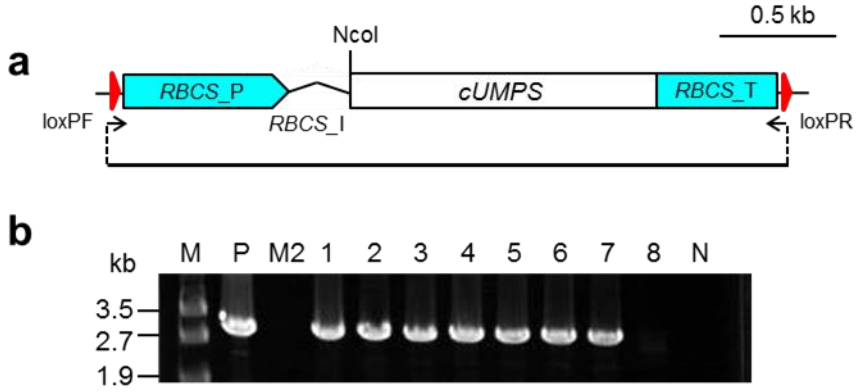

Supplementary Figure S2. PCR detection of the *loxP_cUMPS_loxP* sequence in Ura+ transformants of strain M2 obtained with the ploxP_cUMPS plasmid

a. The map of *loxP_cUMPS_loxP* in the ploxP_cUMPS plasmid. The sites of primer hybridization for amplification of the *loxP_cUMPS_loxP* sequence are shown below the map. Abbreviations: *RBCS*_P, the promoter region of *RBCS*; *RBCS*_I, the first intron of *RBCS*; *cUMPS*, *UMPS* cDNA; *RBCS*_T, the terminator region of *RBCS*.

b. Agarose gel electrophoresis of the PCR-amplified *loxP_cUMPS_loxP* fragments amplified from genomic DNAs. PCR screening revealed positive amplification of the intact *loxP_cUMPS_lox*P sequence in 40 out of 436 Ura+ transformants. Seven positive (lane #1 to 7) and one negative Ura+ transformant (lane #8) were selected to prepare this electropherogram. Lane M, DNA marker (λ-*Eco*T14 I digest) with molecular sizes in bp. DNA templates were: lane P, ploxP_cUMPS plasmid DNA; lane M2, genomic DNA of strain M2; lanes 1–8, genomic DNA of Ura+ transformants of strain M; and lane N, no template.

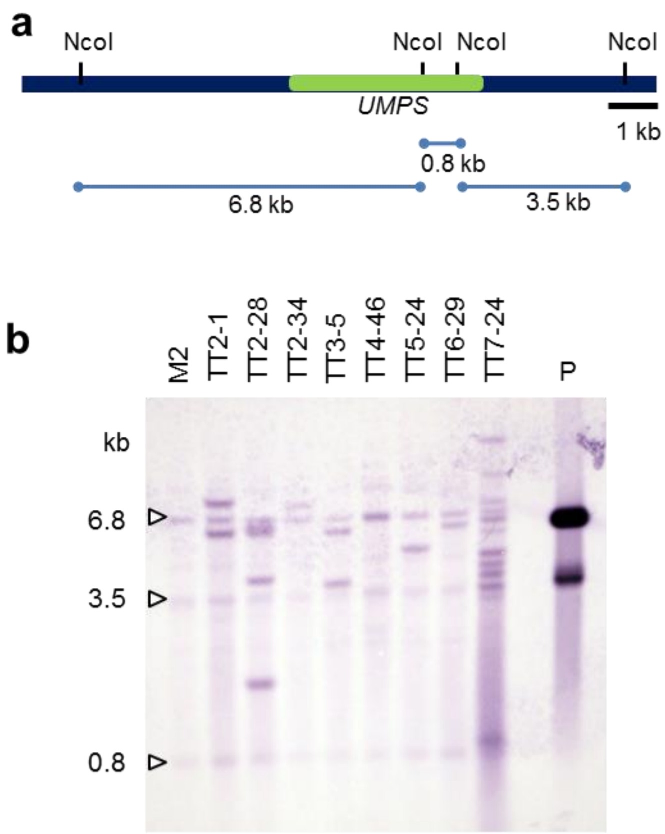

Supplementary Figure S3. Analysis of *cUMPS* copy number by Southern blotting in Ura$^+$ transformants of strain M2 obtained with the ploxP_cUMPS plasmid

a. The NcoI restriction sites in the 13-kb genomic DNA region comprising the endogenous *UMPS* gene.

b. Southern blotting of NcoI-digested genomic DNAs of indicated strains with a digoxigenin-labeled *cUMPS*. Lane M2, strain M2; next eight lanes, Ura$^+$ transformants obtained with the ploxP_cUMPS plasmid whose names are indicated above the lanes; lane P, the ploxP_cUMPS plasmid.

a

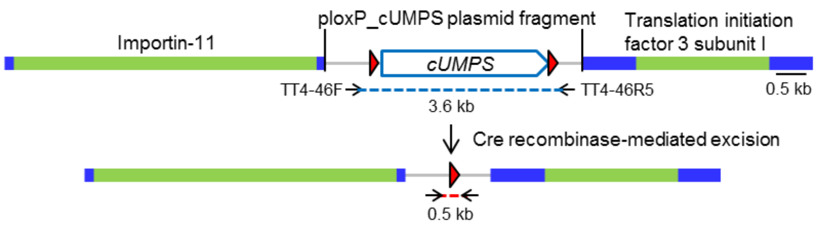

b

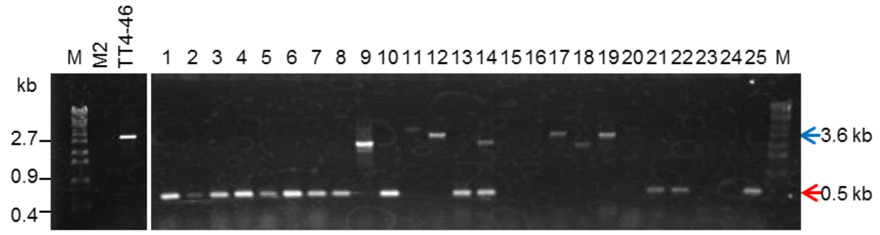

c

GCTCTTCCAAGAAAACAGTGTCCAACAGTGCTACCGTTGTCATTGGCCGAACATAGTTGTCTGTTAGGGGAACCCCCGCTCACGGTAGGCAGGCA
GCCCAATTCGCCCTATAGTGAGTCGTATTACAATTCACTGGCCGTCGTTTTACAACGTCGTGACTGGGAAAACCCTGGCGTTACCCAACTTAATC
GCCTTGCAGCACATCCCCCTTTCGCCAGCTGGCGTAATAGCGAAGAGGCCCGCACCGATCGCCCTTCCCAACAGTTGCGCAGCCTGAATGGCGAA
TGGAGGCCCGCACCGATCGCCCTTCCCAACAGTTGCGCAGCCTGAATGGCCACCTGACGCGCCCTGTAGCGGCGCATTAAGCGCGGCGGGTGTGG
TGGTTACGCGCAGCGTGACCGCTACACTTGCCAGCGCCCTAGCGCCCGCTCCTTTCGCTTTCTTCCCTTCCTTTCTCGCCACGTTCGCCGGCTTT
CCCCGTCAAGCTCTAAATCGGGGGCTCCCTTTAGGGTTCCGATTTAGTGCTTTACGGCACCTCGACCCCAAAAAACTTGATTAGGGTGATGGTTC
ACGTAGTGGGCCATCGCCCTGATAGACGGTTTTTCGCCCTTTGACGTTGGAGTCCACGTTCTTTAATAGTGGACTCTTGTTCCAAACTGGAACAA
CACTCAACCCTATCTCGGTCTATTCTTTTGATTTATAAGGGATTTTGCCGATTTCGGCCTATTGGTTAAAAAATGAGCTGATTTAACAAAAATTT
AACGCGAATTTTAACAAAATA**TAACGCTTACAATTTCCATTCGCC**ATTCAGGCTGCGCAACTGTTGGGAAGGGCGATCGGTGCGGGCCTCTTCGC
TT4-46F
TATTACGCCAGCTGGCGAAAGGGGGATGTGCTGCAAGGCGATTAAGTTGGGTAACGCCAGGGTTTTCCCAGTCACGACGTTGTAAAACGACGGCC
AGTGAATTGTAATACGACTCACTATAGGGCGAATTGGGTACCGGGCCCCCCCTCGAGGTCGACGGTATCGATAAGCTTGATATCGAATTCATAAC
TTCGTATAGCATACATTATACGAAGTTATATGCCCTCAATCGGCCGATTTCATGCATGAAGAAGAAGATGATGATTACACATCATTCCAGCCATC
TAGGGCTGCCATCAGTAATTGCATGACCCGCAACTTCCCAGTCAGTCAAATGCACAATAGAAGCATAGTTGCATGGGAATCAACCCAGCAGCCTC
CCGCAGAGCTAGCCTAGGTACGTGTACGCAGCCACATAATCATCATGAATGATTCTGCACGGTGCTATGATAGCAACAGATACTCGCCAGTGCGC
ACGCAAGCAATCTCATCACACCACTGTGGTCAGGCAAGCACGATATAGATGTCAAATGACAGCTCCATTGGGAGTGCAAGTCTACTAGAGTGCAA
GCCTACTAGTAGTAGGAAGGTCCAGCGCATGCCAAAACTCCTGCTAAGGGGTTGCCCAAGCCCCTTGGCTGGCCAAAAGTCCTTAATATTTTGCT
AGGTAAAGGGGATCTTCCCGGAGGTGGTGTTGATGAACATTTGTACACCAGCATCCGGGTCCTTTTGGACTCTGAAAACGTGAGCGAGCAGCGAC
TGCTTGCAGCTTCCAAACGAAAGGTCTGGAAGCGCTGGAGTGGTGAGTCAGGGGTGACTCAGCAAACACCAAGGACACACGTTTTCACCCTACCC
TCAGAACCCTTGAAATAGGGGTCGATTTCCCTCTAACTTAAGTGGTTATCCACAAACTCACGACGATAAGAGCCAGCGCCAGCACCATTTCGTTC
CTCATTTTCAGCTGAAAGCAAAGCCTTTGCATACTTATCGCTCTCAGATTACAATCGCAATGGGTACGCAGCTTTCTCACTCCCTCACCGCGTGT
GGTTCACTCAGGTTTCTGGCTAAGGAAAGTCTGATGTGACTTTCCTTAGACGTTAAAGCAGAATCACCCCATGCTTTAAGCAGTTTTATGTGTCT
TGCCCTTCATGTGAAGGGTCTGTACTGTTGCAGAGTGCTCTCTTGTGTTCTTGAGTGTTCTTGTCTCCAGTGTGCCCATTAACTTTATCCATGTC
TTATGTGTCCAGGACTGCGCCAAAGAAACTGACGCTGCGCTGTTTTGCTTTGTGCAGCCATGGCAACTTCAACTCCCTCAGTGGCCACAGCTGCG
GAGCTGAAACCAAAGGAAGGGCCTATCACGTCACAGGAGTTTGAAGAGCTCGTGCTGCGTTTGCATGAAATCGAGGCTGTCAAATTCGGCAACTT
CAAGCTCAAGAGCGGGCTCATGTCGCCGATCTACATCGACCTGCGCGTCATCGTGTCCTATCCGGACGTCCTGCGCAGGGTGGCGGAGGTGATGT
GGCATCAGGTGTCGGGCGCTGCATTTGATGTGATGTGTGGCGTGCCGTACACGGCGCTGCCCATAGCCACATGCATGTCGCTGCTGCACGGCACG
CCCATGCTGATGCGGCGCAAGGAGGTCAAGGAGTACGGCACCAAGAAGGCCATTGAGGGCGCCTTCAAGAAGGGCCAGACCTGCCTCATTGTGGA
GGACCTGGTCACCAGCGGCGCCTCCGTCATGGAGACTGTGGAGCCCCTCGAGGTGGAGGGGCTGAAGGTGACGGATGTGGTGGTGCTCATAGACC
GGGAGCAGGGCGGCGCGGCGCGCATGGCCAGCAACGGGCTGCGACTGCACTCCGCGTTCACGCTCTCCTTCATCATCAAGACGCTGCAGGCGCAC
GGCCTTGTCAGCGCTAAGGTGGCCGACAGCGTCGCTGCCTTCATAGCCGCCAACCAGACCTTCACCCCCTCTGCAGCCGCCCCCACACCCTCACC
TGCCGCTCCTGCACAGCCCAAACGGCTGCCATTTGAGGAGCGCGCAAGCCTATGTCAGAACGCAGCCGGCAGGAAGCTGTTGGAGCTGATGGCGC
GCAAGCGGACCAACCTGGCGGTGGCGGCGGACGTGGCGACGGTGGAGGAGATGCTACGCATTGCGGACGCGGCCGGGCCACACATCGCCGTCTTC
AAGACGCACGTGGACATCTTCGACAAGTGGGATGATGGCATCGCCACCCAGCTGCGCCATCTGGCTGACAAGCATGAGTTCTTGATATTCGAGGA
CAGGAAGTTTGCGGACATCGGTAACACAGTGGTGTCGCAGTACGGCGGAGGCATCTACAAGATTGCCGACTGGTCAGACATCACCAATGCACACC
TGGTCCCTGGGCCAGGCATCATCGATGGTCTCCGTAAGGTGGGCCAGGAGAAGGGGCGCGGCCTGCTGTTGCTGGCTGAGATGAGCTCCAAGGGA
GCCCTTGCCACAGGGGCCTACACCGAGAAGGTTGCTGAAGCGGCAGCTGCCAACCAGGATTTTGTCATGGGCTTCATCTGCCAGTCACCAGCCAA
GTGGGCCACACCGGTCCCTCCAGGTCTGGTGCACATGACACCGGGAGTGCAGCTAGCGTCTGGGAGCGACGCATTGGGGCAGCAGTACAACACAC
CTGCTTCTGTCATCGGCCAGGGCGGCAGCGATGTCATCATCGTGGGGAGGGGAATCATTAAGGCGGCAGACCCCGCTGCAGCGGCCGCACAGTAC
AGAGAAGCCGGCTGGGCCGCCTATGAGGCCACGCTGGCATGAAGCTTATCGATACCGTCGACAGGTTCTAAGCAATGACCTGGGCTGTACATCAT
TAGCAAACTGAATCTGCAGCTTTCTAGGCTCTTGCTGTTCCTAGGAACTTGGGGTTCAAGACTGGGTTTTTGCTGTGGTTCCTGACTGCAGACCA
TCACTGTCTGGGTTGTCGTGTCTAATGTCCATAGGTGTGAGCACATGCTCCACTTCAATTCCTACTGCTGGGTAACTGGCGCTGTCTGAGGGACT
TCTGAGAAGAATGCCGAGAAGAACTTGAGCGATTTGTAAGGGGATATTTTGGGTCTGTACCAAAGGCCCAGACTGGGCTTCCAGTGCATCGCACA
TATGCATCTGCATGTCCATTCATACAATCAACCTGTTGACTGCATGACCCAGATCACAACCTCCTTGAGTTGGAAGTAGCTTCTTCTACAGATAG
AAAATGGTGCATGTCAGCTGAGGGTTGCAGGCGTTTAAAAGTGCATCAAGTTTGCAGAACACATGGTGATTCATGACTGCATGGAAACAGACCAG
CACAATAACTTCGTATAGCATACATTATACGAAGTTATGGATCCACTAGTTCTAGAGCGGCCGCCACCGCGGTGGAGCTCCAGCTTTTGTTCCCT
TTAGTGAGGGTTAATTTCGAGCTTGGCGTAATCATGGTCATAGCTGTTTCCTGTGTGAAATTGTTATCCGCTCACAATTCCACACAACATACGAG
CCGGAAGCATAAAGTGTAAAGCCTGGGGTGCCTAATG**AGTGAGCTAACTCACATTAATTG**CGTTGCGCTCACTGCCCGCTTTCCAGTGAGCGCAG
TT4-46R5
CTTTCTCACTCCCTCCCCTCCCCTCCCTCCATGTCGCCGATCGACATGAGCCCGCTCTTGAGCTGAAGTGCCGAATTTTTGGAAGCTGCAAGCAG

3.6-kb PCR fragment

d

TAACGCTTACAATTTCCATTCGCCATTCAGGCTGCGCAACTGTTGGGAAGGGCGATCGGTGCGGGCCTCTTCGCTATTACGCCAGCTGGCGAAAGGGGGAT
TT4-46F
GTGCTGCAAGGCGATTAAGTTGGGTAACGCCAGGGTTTTCCCAGTCACGACGTTGTAAAACGACGGCCAGTGAATTGTAATACGACTCACTATAGGGCGAA
TTGGGTACCGGGCCCCCCCTCGAGGTCGACGGTATCGATAAGCTTGATATCGAATTCATAACTTCGTATAGCATACATTATACGAAGTTATGGATCCACTA
GTTCTAGAGCGGCCGCCACCGCGGTGGAGCTCCAGCTTTTGTTCCCTTTAGTGAGGGTTAATTTCGAGCTTGGCGTAATCATGGTCATAGCTGTTTCCTGT
GTGAAATTGTTATCCGCTCACAATTCCACACAACATACGAGCCGGAAGCATAAAGTGTAAAGCCTGGGGTGCCTAATG**AGTGAGCTAACTCACATTAATTG**
TT4-46R5

Supplementary Figure S4. **PCR analysis to detect Cre-recombinase-mediated excision of the *loxP_cUMPS_loxP* sequence**

a. A 4.4-kb DNA fragment consisting of the *loxP_cUMPS_loxP* sequence flanked by partial vector sequences was shown to be integrated in the intergenic region between the gene for predicted importin-11 (*IPO11*) and that for translation initiation factor 3 subunit I (*EIF3I*). The two green boxes indicate transcribed regions, while blue boxes intergenic regions. The *loxP* sites are indicated by red triangles. Two arrows beneath the map denote the PCR primer set (TT4-46F and TT4-46R) used for the amplification of either a 3.6-kb fragment from genomic DNAs without the excision event or a 0.5-kb fragment from genomic DNAs which underwent Cre recombinase-mediated excision.

b. Agarose gel electrophoresis of the 3.6-kb or 0.5-kb fragments amplified from genomic DNAs of indicated strains. Lane M, DNA size marker (λ-EcoT14 I digest) with molecular sizes in bp. DNA templates were: lane M2, genomic DNA of strain M2; lane TT4-46, genomic DNA of strain TT4-46; lanes 1–25, genomic DNA of 5-FOA^r derivatives of strain TT4-46 isolated after intracellular delivery of Cre recombinase.

c. Nucleotide sequence of chromosome region containing the *loxP_cUMPS_loxP* sequence integration site. Partial genomic DNA of strain Obi is shown as blue letters, partial vector DNA as black letters, *loxP* DNA as red letters, and *cUMPS* expression cassette DNA as green letters. The arrows indicate the positions and sequences of PCR primers TT4-46F and TT4-46R5.

d. Nucleotide sequence of 0.5-kb PCR fragment. The color codes used are the same as those used in **c**.

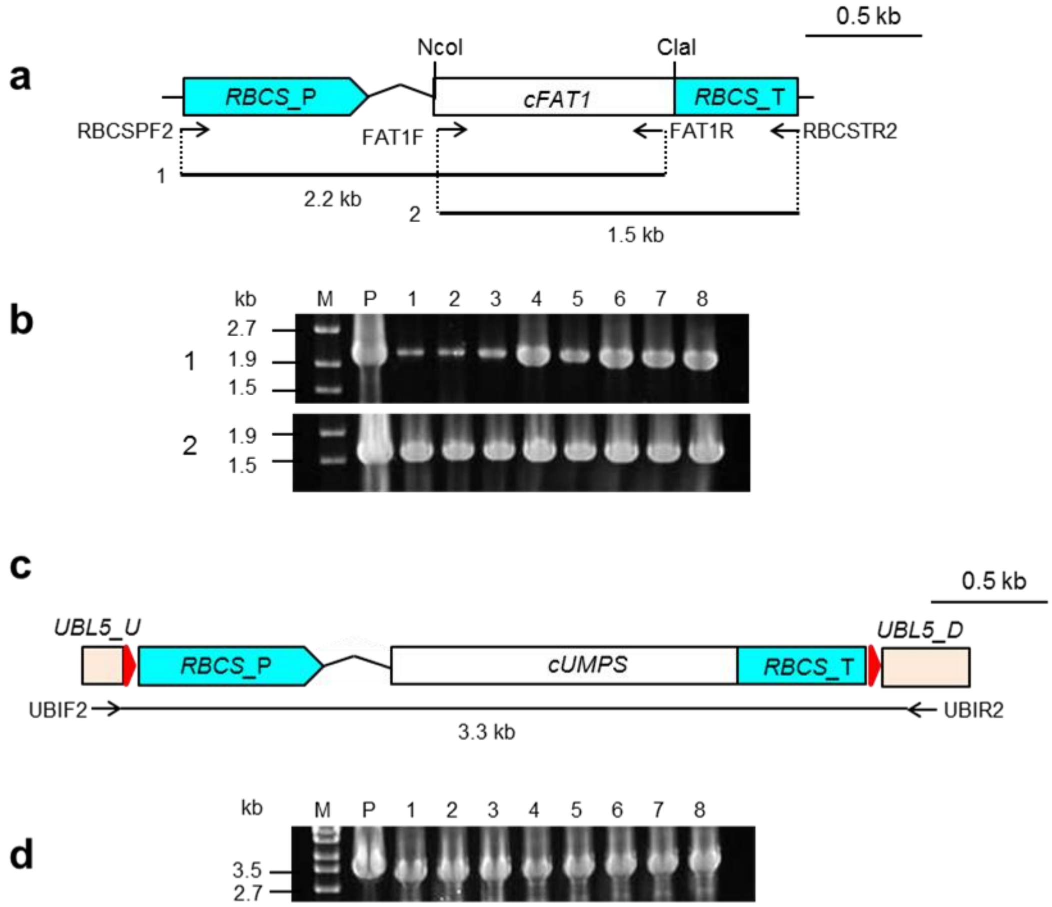

Supplementary Figure S5. PCR detection of the *cFAT1* expression cassette sequence and the *loxP_cUMPS_loxP* sequence in Ura$^+$ transformants of strain M2

a. The structure of the *cFAT1* expression cassette and the sizes of the PCR products (1 and 2) amplified using two different primer sets.

b. Agarose gel electrophoresis analyses of PCR products (1 and 2). The *cFAT1* expression cassette DNA and the *loxP_cUMPS_loxP* DNA were used to transform strain M2. Among 312 Ura$^+$ transformants, eight transformants named Fox023, Fox0224, Fox0232, Fox0329, Fox0929, Fox1115, Fox1117, and Fox1120 carried intact *cFAT1* expression cassette sequence, and these strains were selected to prepare this electropherogram. Panels 1 and 2 show the detection of PCR products 1 and 2,

respectively. Lane M: DNA marker (λ-*Eco*T14 I digest) with molecular sizes in bp. DNA templates were: lane P, pFAT1 plasmid DNA which carries the *cFAT1* expression cassette; lanes 1–8, genomic DNAs of Fox023, Fox0224, Fox0232, Fox0329, Fox0929, Fox1115, Fox1117, and Fox1120.

c. The partial structure of the ploxP_cUMPS _2 plasmid in which the *loxP_cUMPS_loxP* sequence is inserted within the *UBL5* sequence. *UBL5_U* and *UBL5_D* are upstream and downstream regions, respectively, of *UBL5*. Primer-hybridization sites for amplification of the *loxP_cUMPS_loxP* sequence are shown below the map.

d. Agarose gel electrophoresis analyses of 3.3-kb PCR products. Lane M, DNA marker (λ-*Eco*T14 I digest) with molecular sizes in bp. DNA templates were: lane P, ploxP_cUMPS_2 plasmid DNA; lanes 1–8, genomic DNAs of Fox023, Fox0224, Fox0232, Fox0329, Fox0929, Fox1115, Fox1117, and Fox1120.

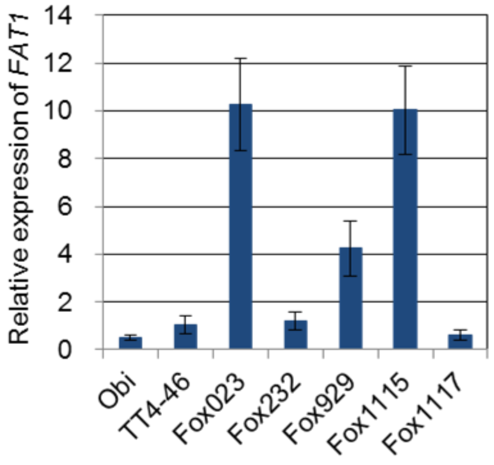

Supplementary Figure S6. Expression of *cFAT1* in Ura$^+$ transformants of strain M2

RT-qPCR detected transcripts of both endogenous *FAT1* and transgenic *cFAT1*. 18S rRNA was used as an internal control. The data are presented as relative expression levels of *FAT1* normalized to 18S rRNA expression in transgenic strains taking the expression level in strain Obi as 1. Bars represent the standard deviation of three replicates.

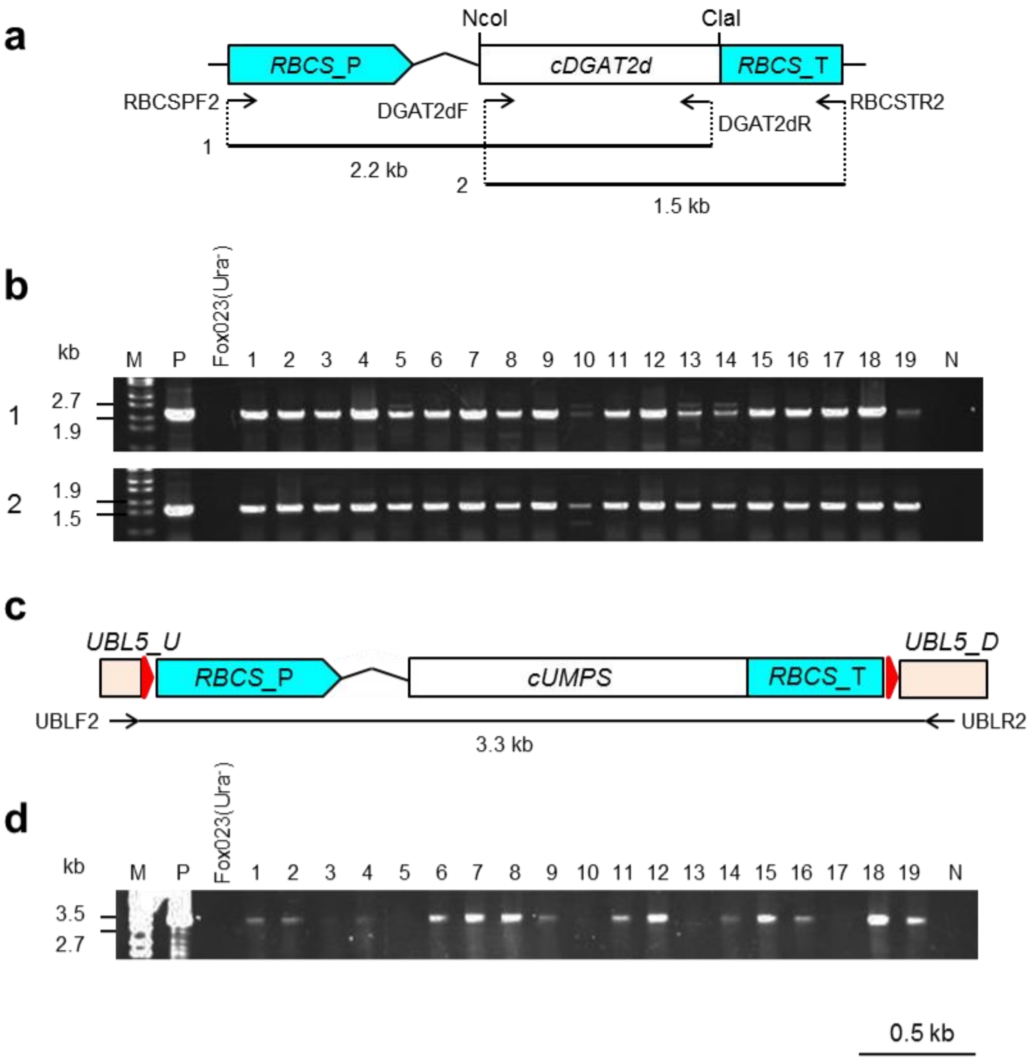

Supplementary Figure S7. PCR detection of the *cDGAT2d*-expression-cassette sequence and the *loxP_cUMPS_loxP* sequence in Ura^{+} transformants of strain Fox023(Ura^{-})

a. The structure of the *cDGAT2d* expression cassette and the sizes of the PCR products (1 and 2) amplified using two different primer sets.

b. Agarose gel electrophoresis analyses of PCR products (1 and 2). Among 263 Ura^{+}

transformants of strain Fox023(Ura$^-$),19 strains carried the intact *cDGAT2d* expression cassette. These strains were selected to prepare this electropherogram. Panels 1 and 2 show the detection of PCR products 1 and 2, respectively. Lane M, DNA marker (λ-*Eco*T14 I digest) with molecular sizes in bp. DNA templates were: lane P, pDGAT2d plasmid DNA; lane Fox023(Ura$^-$), genomic DNA of strain Fox023(Ura$^-$); lanes 1–19, genomic DNA of Ura$^+$ transformants of strain Fox023(Ura$^-$) carrying the intact *cDGAT2d* expression cassette; lane N, no template.

c. The structure of the *loxP_UMPS_loxP* cassette flanked by the 5' and 3' regions of the ubiquitin-like protein 5 gene (*UBL5*). Primer-hybridization sites for the amplification of the *loxP_cUMPS _loxP* sequence are shown below the map.

d. Agarose gel electrophoresis analyses of 3.3-kb PCR products amplified from genomic DNAs of indicated strains. Lane M, DNA marker (λ-*Eco*T14 I digest) with molecular sizes in bp. DNA templates were: lane P, ploxP_cUMPS_loxP2 plasmid DNA; lane Fox023(Ura$^-$), strain Fox023(Ura$^-$); lanes 1–19, the same transformants as those in **b**; lane N, no template.

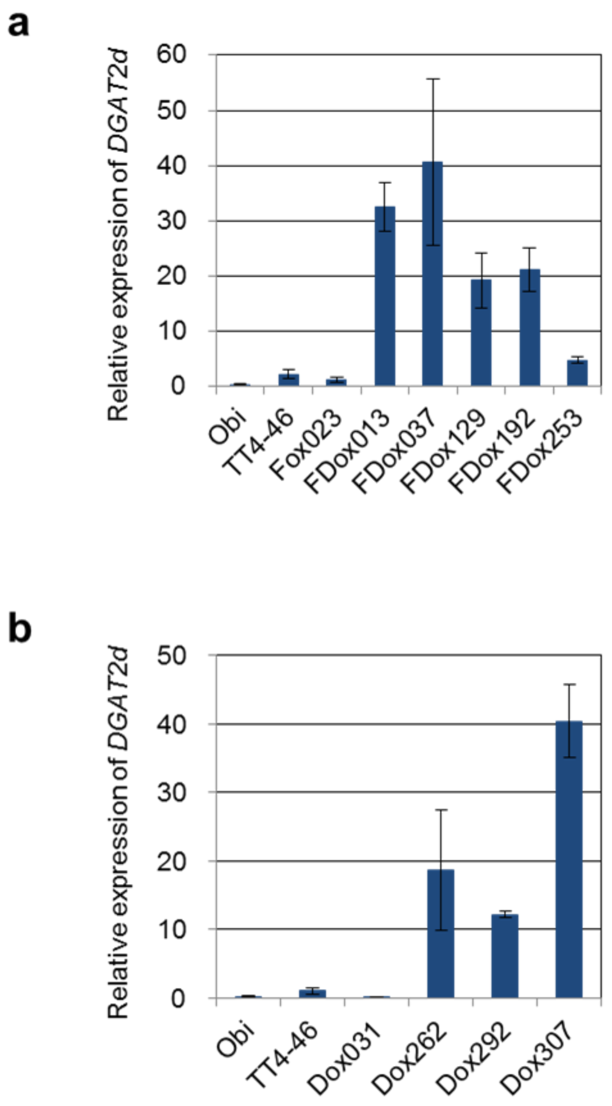

Supplementary Figure S8. Expression of *cDGAT2d* in Ura^{+} transformants of strains Fox023(Ura^{-}) and M2

a. RT-qPCR analysis of the expression of c*DGAT2d* in strains derived from strain Fox023(Ura^{-})

b. RT-qPCR analysis of the expression of c*DGAT2d* in strains derived from strain from strain M2 (b).

In **a** and **b**, RT-qPCR detected transcripts of both endogenous *DGAT2d* and transgenic

cDGAT2d. 18S rRNA was used as an internal control. The data are presented as relative expression levels of *DGAT2d* normalized to 18S rRNA expression in transgenic strains taking the expression level in strain Obi as 1. Bars represent the standard deviation of three replicates.
